# Maternal H3K27me3 controls DNA methylation-independent genomic imprinting

**DOI:** 10.1038/nature23262

**Published:** 2017-07-19

**Authors:** Azusa Inoue, Lan Jiang, Lu Falong, Tsukasa Suzuki, Yi Zhang

**Affiliations:** 1 Howard Hughes Medical Institute, Boston Children’s Hospital, Boston, Massachusetts 02115, USA; 2 Program in Cellular and Molecular Medicine, Boston Children’s Hospital, Boston, Massachusetts 02115, USA; 3 Division of Hematology/Oncology, Department of Pediatrics, Boston Children’s Hospital, Boston, Massachusetts 02115, USA; 4 Department of Genetics, Harvard Medical School, Boston, Massachusetts 02115, USA; 5 Harvard Stem Cell Institute, WAB-149G, 200 Longwood Avenue, Boston, Massachusetts 02115, USA

**Keywords:** Allelic chromatin accessibility, Allelic gene expression, Germline DNA methylation, Allelic H3K27me3, H3K27me3-dependent genomic imprinting, Mouse early development

## Abstract

Mammalian sperm and oocytes have different epigenetic landscapes and are organized in different fashion. Following fertilization, the initially distinct parental epigenomes become largely equalized with the exception of certain loci including imprinting control regions (ICRs). How parental chromatin becomes equalized and how ICRs escape from this reprogramming is largely unknown. Here we profiled parental allele-specific DNase I hypersensitive sites (DHSs) in mouse zygotes and morula embryos, and investigated the epigenetic mechanisms underlying allelic DHSs. Integrated analyses of DNA methylome and H3K27me3 ChIP-seq data sets revealed 76 genes with paternal allele-specific DHSs that are devoid of DNA methylation but harbor maternal allele-specific H3K27me3. Interestingly, these genes are paternally expressed in preimplantation embryos, and ectopic removal of H3K27me3 induces maternal allele expression. H3K27me3-dependent imprinting is largely lost in the embryonic cell lineage, but at least 5 genes maintain their imprinting in the extra-embryonic cell lineage. The 5 genes include all previously identified DNA methylation-independent imprinted autosomal genes. Thus, our study identifies maternal H3K27me3 as a DNA methylation-independent imprinting mechanism.

Sperm and oocytes are generated from primordial germ cells through distinct processes. Consequently, their genomes are packaged differently with distinct epigenetic landscapes ^1^. Following fertilization, paternal chromatin releases protamines and is repackaged with maternally-stored histones that are devoid of most histone modifications, while maternal chromatin harbors various histone modifications inherited from oocytes ^2,3^. The different processes of parental chromatin formation result in parental epigenetic asymmetry in zygotes ^1^, which becomes largely equalized during subsequent development with the exception of certain genomic loci, including ICRs ^4^.

Transcriptional regulatory elements, such as promoters and enhancers, can be mapped by DNase I hyper-sensitivity assay ^5,6^. By using a low-input DNase I-sequencing (liDNase-seq) technique, we recently mapped the transcriptional regulatory landscape of preimplantation embryos and SNP-based analysis revealed that chromatin accessibility of the two parental alleles is overall comparable except imprinted gene promoters ^7^. A similar conclusion was also reached using an assay for transposase-accessible chromatin with high throughput sequencing (ATAC-seq) ^8^. However, the mechanisms underlying parent-of-origin specific chromatin accessibility are unknown.

## Allelic DHSs in zygotes

**Extended data figure 1.**
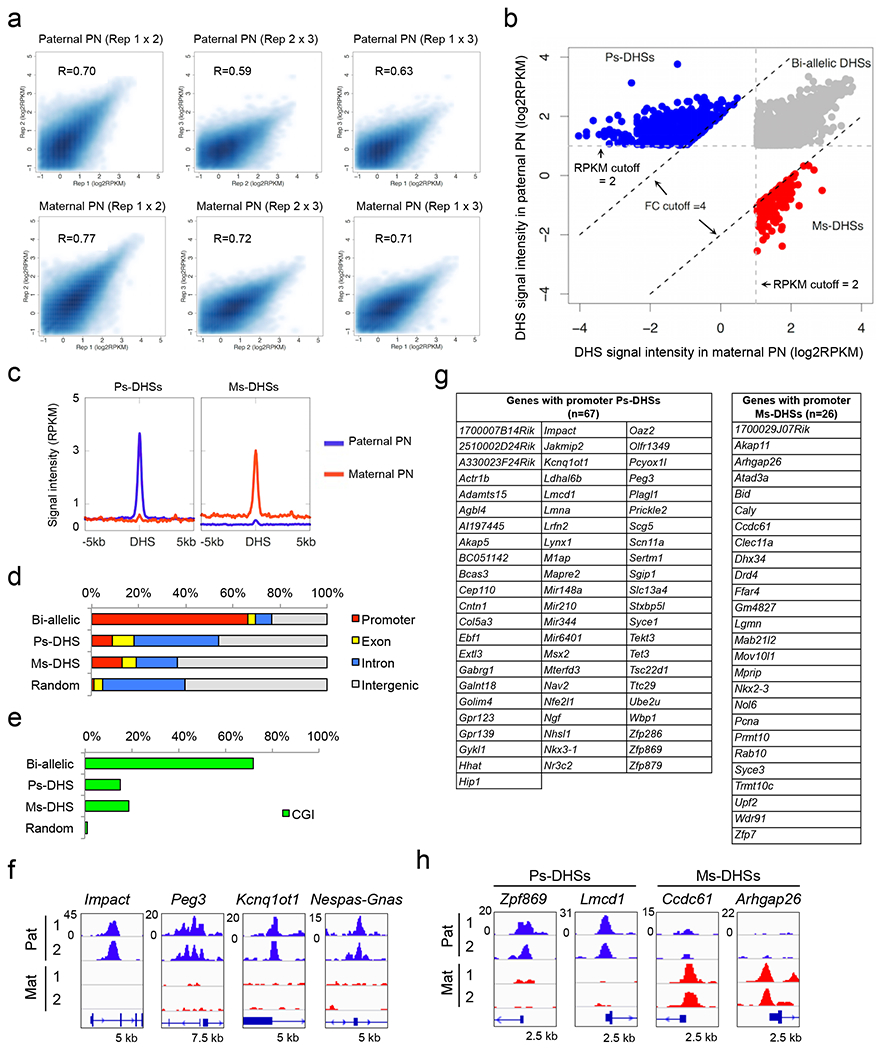
Identification of parental allelic DHSs, related to Figure 1 **a**, Scatter plots showing the correlation of DHSs between three biological replicates in paternal and maternal pronuclei (PN). **b**, Scatter plot showing bi-allelic DHSs (gray), Ps-DHSs (blue), and Ms-DHSs (red). The cutoffs used to define these DHS groups are indicated. **c**, Averaged DHS signals of Ps-DHSs and Ms-DHSs within ± 5 kb around DHSs. **d**, Genomic distribution of DHSs. Promoters represent ± 1 kb around TSSs. Random indicates the percentages of each genomic element of the mouse genome. **e**, Percentages of DHSs located at CpG islands (CGIs). Promoters represent ± 1 kb around TSSs. The genomic locations of CGIs is defined previously ^9^ **f**, Genome browser view of Ps-DHSs at imprinting control regions (ICRs) of representative imprinted genes. The genomic locations of ICRs were referred in Kobayashi et al., 2012 ^9^. **g**, List of genes harboring promoter Ps-DHSs or Ms-DHSs in zygotes. **h**, Genome browser view of representative allelic DHSs at gene promoters not previously known to be imprinted.

**Figure 1.**
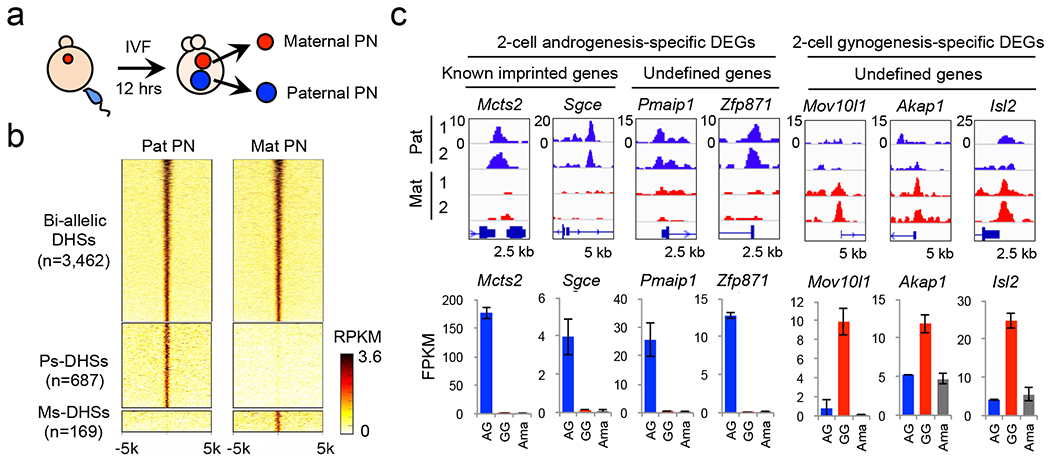
Allelic DHSs in zygotes mark allelic gene expression at ZGA **a**, Schematic for identifying parental allele-specific DHSs in zygotes. IVF, *in vitro* fertilization. PN, pronucleus. **b**, Heat map showing bi-allelic, paternal allele-specific (Ps-DHSs), and maternal allele-specific DHSs (Ms-DHSs) in zygotes. Each row represents liDNase-seq signal intensity at a DHS ± 5 kb. **c**, Representative androgenesis (AG)- and gynogenesis (GG)-specific differentially expressed genes (DEGs) harboring allelic promoter DHSs in zygotes. Upper panels are genome browser views of DHSs in paternal and maternal pronuclei with biological duplicates. The DHS signal intensity and the genomic length of each view (kb) are indicated at the upper left and the bottom of each panel, respectively. Lower graphs are gene expression levels in AG, GG and α-amanitin-treated (Ama) 2-cell embryos. Error bar, standard deviation of biological duplicates. Note that GG-specific expression of *Akap1* and *Isl2* is evident after subtraction of maternal pool transcripts.

To comprehensively profile parental allele-specific DHSs in zygotes, we isolated paternal and maternal pronuclei from PN5-stage zygotes and performed liDNase-seq (Fig. 1a, Extended Data Fig. 1a). Using stringent criteria (Extended Data Fig. 1b) and excluding data of sex chromosomes, we identified 3,462, 687, and 169 of bi-allelic DHSs, paternal allele-specific DHSs (Ps-DHSs), and maternal allele-specific DHSs (Ms-DHSs), respectively (Fig. 1b, Extended Data Fig. 1c and Table S1). The genomic location of allelic DHSs is heavily biased to non-promoter elements when compared to bi-allelic DHSs that are enriched in promoters and CpG islands (Extended Data Fig. 1d, e). Similar to previous finding ^7^, Ps-DHSs include ICRs of known imprinted genes (Extended Data Fig. 1f). Interestingly, both Ps- and Ms-DHSs also include promoters of genes previously not known to be imprinted (Extended Data Fig. 1g, h).

**Extended data figure 2.**
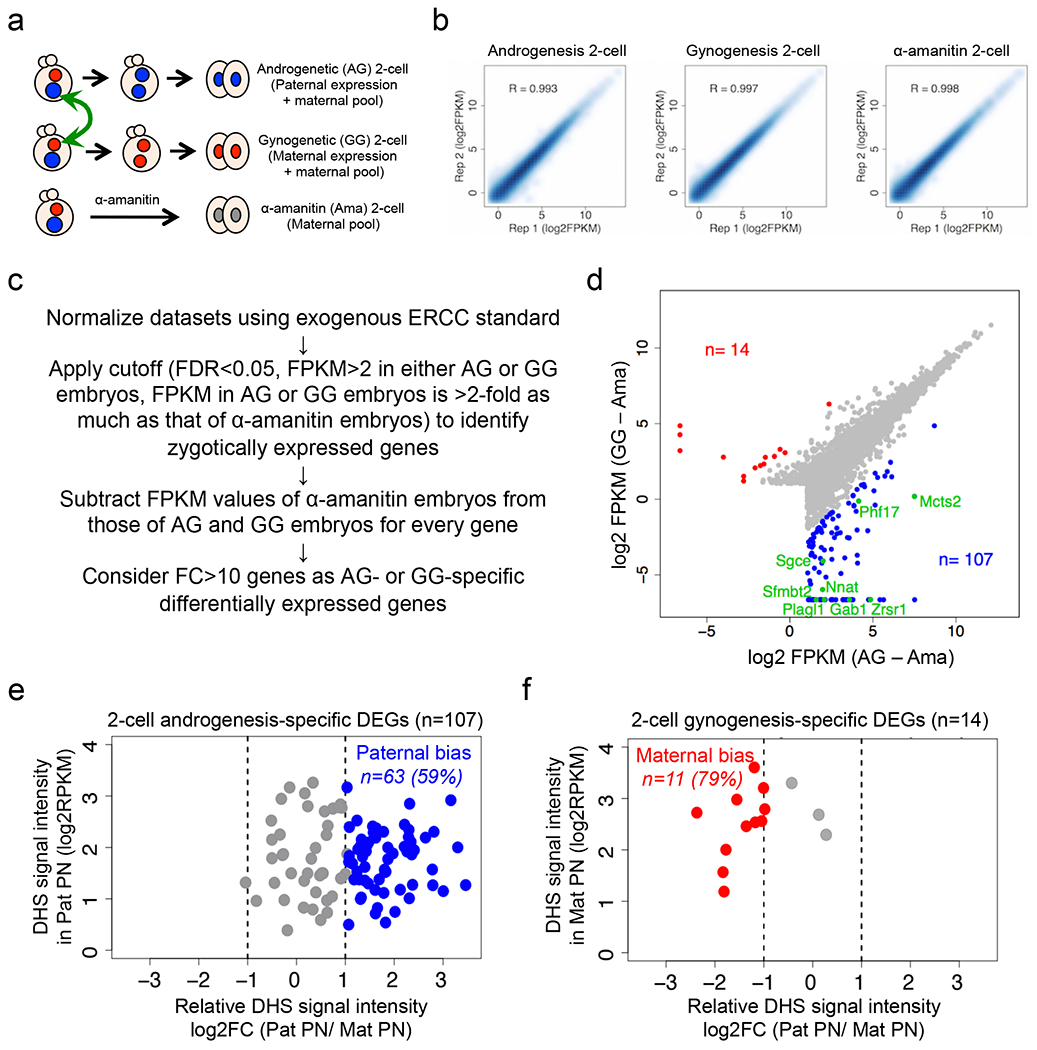
Experimental scheme, RNA-seq reproducibility and analysis scheme, related to Figure 1 **a**, Schematic for identifying parental allele-specific gene expression at ZGA. Androgenetic (AG) embryos and gynogenetic (GG) embryos were produced by pronuclear transfer. AG 2-cell embryos contain paternally-expressed nascent transcripts and maternally-stored transcripts. GG 2-cell embryos contain maternally-expressed nascent transcripts and maternally-stored transcripts. α-amanitin-treated (Ama) 2-cell embryos contain maternally-stored transcripts only. **b**, Scatter plot showing the correlation between biological duplicate of 2-cell RNA-seq samples. **c**, Flowchart for avoiding maternally-stored transcripts and identifying nascent allelic transcripts at ZGA. **d**, Scatterplot of nascent transcripts in AG and GG 2-cell embryos. For each gene, the FPKM value in Ama embryos was subtracted from that in AG and GG embryos, respectively. AG- and GG-specific differentially expressed genes (DEGs) (FC>10) are indicated in blue and red, respectively. Known imprinted genes are indicated in green. **e, f,** Scatterplot showing DHS allelic bias at promoters (±0.5 kb at TSS) of androgenesis- (**e**) and gynogenesis- (**f**) specific differentially expressed genes (DEGs). FC>2 was considered as ‘bias’ (blue or red).

Since promoter DHSs can prime gene expression at the next developmental stage ^7^, we asked whether allelic DHSs in zygotes can prime allelic gene expression at zygotic genome activation (ZGA). RNA-seq analysis of 2-cell stage androgenetic (AG) and gynogenetic (GG) embryos, using α-amanitin treatment as a negative control, identified 107 AG- and 14 GG-specific differentially expressed genes (DEGs), including 8 known imprinted genes (Extended Data Fig. 2a–d and Table S2).

Integrated analysis of allelic ZGA and allelic promoter DHSs in zygotes revealed that the majority (59% and 79%) of the AG- and GG-specific DEGs are associated with paternal and maternal allele-biased chromatin accessibility, respectively (Extended Data Fig. 2e, f). Genes showing such a correlation include not only known imprinted genes but also genes not known to be imprinted (Fig. 1c). These results suggest that allelic DHSs in zygotes can mark promoters that are primed for allelic ZGA.

## DNA methylation and allelic DHSs

**Extended data figure 3.**
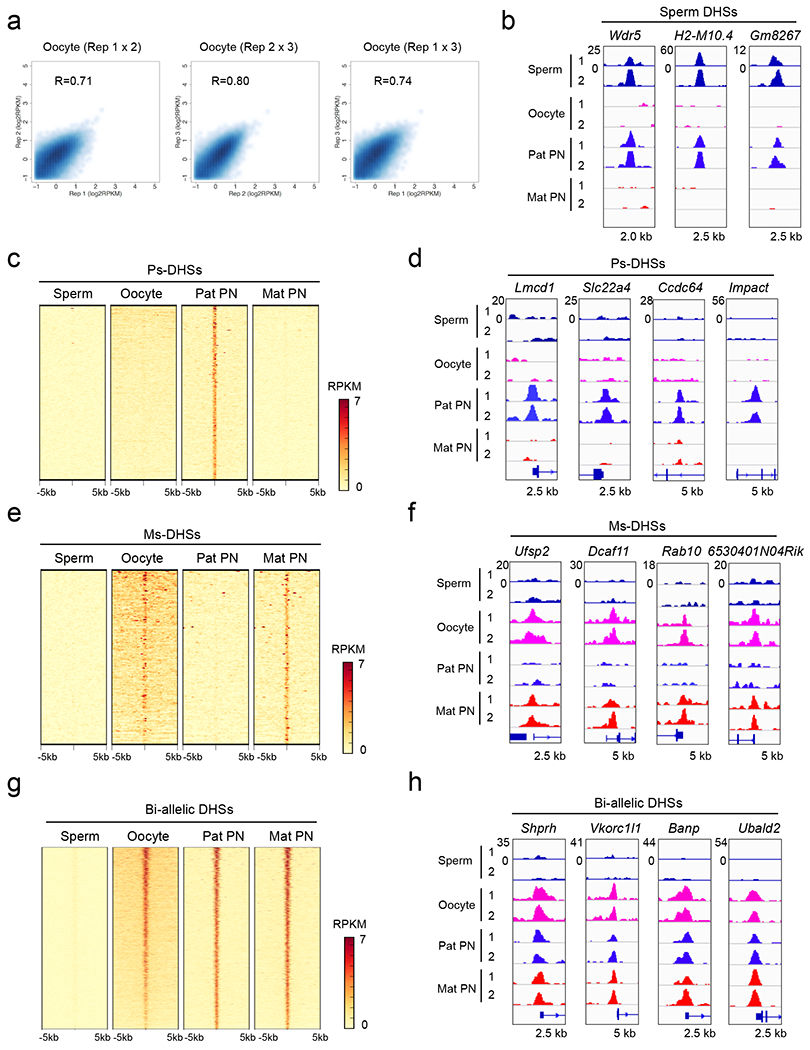
Zygotic Ms-DHSs are inherited from oocyte DHSs, related to Figure 2 **a**, Scatter plot showing the correlation between three biological replicates of liDNase-seq for GV nuclei isolated from fully-grown oocytes. **b**, Genome browser view of sperm DHSs that are passed on to paternal PNs of zygotes. The nearest gene names are indicated at the top of each panel. **c**, Heat map showing Ps-DHSs. Each row represents liDNase-seq signal intensity at a DHS ± 5 kb. Note that Ps-DHSs are largely absent in both sperm or oocytes. **d**, Genome browser view of representative Ps-DHSs. **e**, Heat map showing Ms-DHSs. Note that Ms-DHSs are mostly already present in oocytes. **f**, Genome browser view of representative Ms-DHSs. **g**, Heat map showing biallelic DHSs. **h,** Genome browser view of representative biallelic DHSs.

To understand how allelic DHSs in zygotes are specified, we first examined whether they are inherited from gametes. We profiled DHSs of fully-grown oocytes (Extended Data Fig. 3a) and analyzed sperm DHSs ^7^. Although sperms only have 34 reproducible DHSs (Table S3), some of them contribute to Ps-DHSs (Extended Data Fig. 3b). However, most of Ps-DHSs are absent in sperm and oocytes, indicating that they are generated after fertilization (Extended Data Fig. 3c, d). In contrast, most of Ms-DHSs and bi-allelic DHSs are already present in oocytes (Extended Data Fig. 3e–h), indicating that most maternal DHSs are inherited from oocytes.

**Extended data figure 4.**
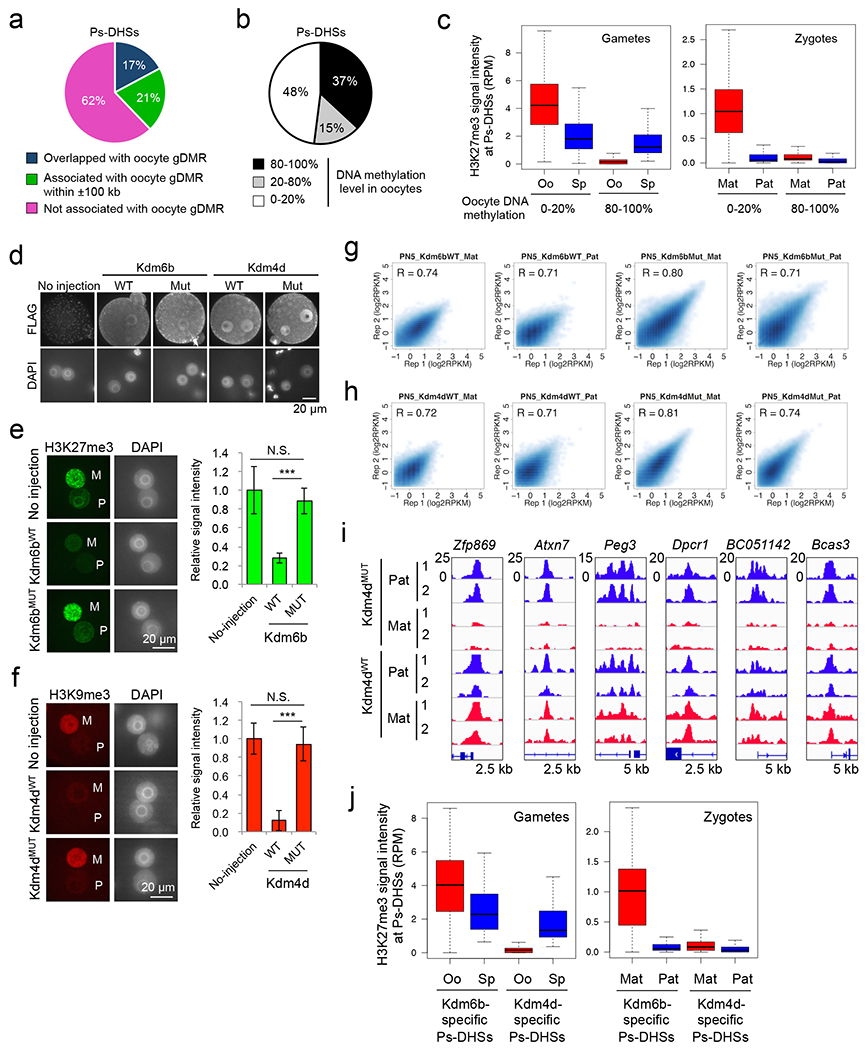
Distinct epigenetic features of Kdm6b- and Kdm4d-affected Ps-DHSs, related to Figure 2 **a**, Pie chart showing percentages of Ps-DHSs that overlap (black) or associated (gray) with oocyte- gDMRs within ±100 kb. Oocyte gDMR was defined by >80% methylation in oocytes and <20% methylation in sperm. **b**, Pie chart showing the percentages of Ps-DHSs organized based on their oocyte DNA methylation levels. **c**, Boxplots showing the H3K27me3 signal levels at Ps-DHSs ±1 kb in gametes (left panel) and zygotes (right panel). Ps-DHSs were divided into oocyte DNA hypomethylated (0-20%, n=296) and hypermethylated groups (80-100%, n=305). Middle lines in the boxes represent the medians. Box edges and whiskers indicate the 25th/75th and 2.5th/ 97.5th percentiles, respectively. **d**, Representative images of *Kdm6b*- or *Kdm4d*-injected zygotes stained with anti-Flag antibody, using non-injected zygotes as negative controls. **e**, Representative images of zygotes stained with anti-H3K27me3 antibody. M, maternal pronucleus. P, paternal pronucleus. The bar graph on the right represents relative immunostaining signal intensity of maternal pronuclei. The averaged signal of non-injected zygotes was set as 1.0. The total numbers of embryos examined were 8 (No injection), 13 (Kdm6b^WT^), and 10 (Kdm6b^MUT^). Error bars indicate SD. ***, *p*<0.001 (two-tailed Student *t*-test). N.S, statistically not significant. **f**, Representative images of zygotes stained with anti-H3K9me3 antibody. The bar graph on right represents relative immunostaining signal intensity in the maternal pronuclei. The averaged signal of non-injected zygotes was set as 1.0. The total numbers of embryos examined were 5 (no-inject), 5 (Kdm4d^WT^), and 7 (Kdm4d^MUT^). Error bars indicate SD. ***, *p*<0.001 (two-tailed Student *t*-test). N.S, statistically not significant. **g**, Scatter plot showing the correlation between biological duplicates of liDNase-seq for maternal (Mat) and paternal pronuclei (Pat) of *Kdm6b*^*WT*^- and *Kdm6b*^*MUT*^-injected zygotes. **h**, Scatter plot showing the correlation between biological duplicates of liDNase-seq for maternal (Mat) and paternal pronuclei (Pat) of *Kdm4d*^*WT*^- and *Kdm4d*^*MUT*^-injected zygotes. **i**, Genome browser view of representative Ps-DHSs affected by Kdm4d^WT^. **j**, Boxplot showing H3K27me3 signals at Kdm6b- or Kdm4d-affected Ps-DHSs ±1 kb in gametes (left panel) and zygotes (right panel). Middle lines in the boxes indicate the medians. Box edges and whiskers indicate the 25th/75th and 2.5th/ 97.5th percentiles, respectively.

To determine how the maternal allele at Ps-DHSs remains inaccessible, we first hypothesized that maternal DNA methylation prevents DHS formation. Analysis of a public whole genome bisulfite sequencing (WGBS) dataset of oocytes and sperm ^9^ revealed that only 17% of Ps-DHSs overlap with oocyte germline differentially methylated regions (gDMRs) (Extended Data Fig. 4a and Table S4). Despite extending to a ±100 kb region flanking Ps-DHSs, only additional 21% are found to be associated with oocyte gDMRs (Extended Data Fig. 4a and Table S4). Even when the oocyte DNA methylation level alone is considered, 48% of Ps-DHSs are devoid of oocyte DNA methylation (Extended Data Fig. 4b), indicating the existence of a DNA methylation-independent mechanism that prevents maternal allelic accessibility.

## Maternal allelic protection by H3K27me3

The fact that Polycomb-mediated H3K27me3 can mediate silencing of DNA hypomethylated promoters ^10^ prompted us to postulate that H3K27me3 might be responsible for maternal allele inaccessibility. Analyses of public ChIP-seq datasets ^11^ revealed that the H3K27me3 level in oocytes is much higher than that of sperm at DNA hypomethylated Ps-DHSs, while it is reversed at DNA hypermethylated Ps-DHSs (Extended Data Fig. 4c**, left**). SNP-tracking analysis revealed that the hypomethylated Ps-DHSs maintain maternal allele-specific H3K27me3 in zygotes (Extended Data Fig. 4c**, right**), indicating that H3K27me3 may be responsible for maternal allele inaccessibility at DNA hypomethylated regions.

**Figure 2.**
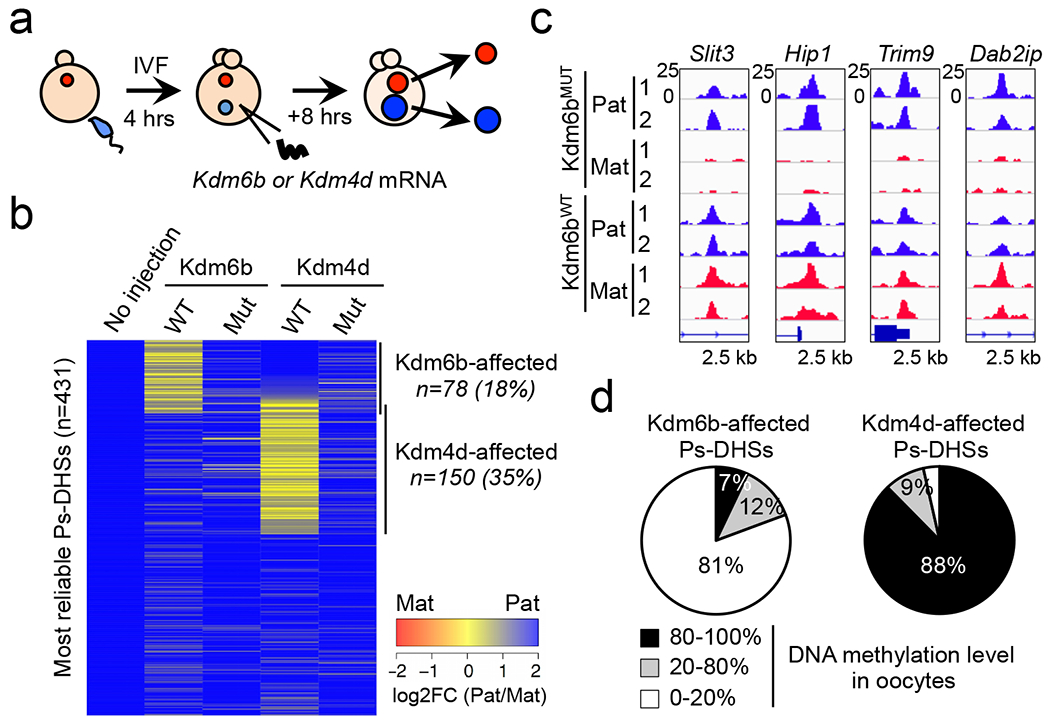
Oocyte-specific H3K27me3 prevents maternal chromatin accessibility at DNA hypomethylated regions **a**, Schematic for studying the role of histone methylations in maternal chromatin inaccessibility. **b**, Heat map showing the allelic bias at Ps-DHSs in *Kdm6b*- or *Kdm4d*-injected zygotes. **c,** Genome browser view of representative Ps-DHSs affected by Kdm6b^WT^. **d**, Pie charts showing Kdm6b- or Kdm4d-affected Ps-DHSs organized based on their oocyte DNA methylation levels.

To test this possibility, we injected mRNA encoding an H3K27me3-specific demethylase *Kdm6b (Kdm6b*^*WT*^) with its catalytic mutant (H1390A) (*Kdm6b*^*MUT*^) as a control ^12^ (Fig. 2a). Similarly, we prepared zygotes injected with an H3K9me3-specific demethylase *Kdm4d* or its catalytic mutant (H189A) ^13^. Both WT and mutant Kdm6b and Kdm4d are expressed at a similar level (Extended Data Fig. 4d), and Kdm6b^WT^ and Kdm4d^WT^, but not their mutants, significantly reduced H3K27me3 and H3K9me3 levels, respectively (Extended Data Fig. 4e, f). LiDNase-seq of isolated pronuclei (Extended Data Fig. 4g, h) revealed that 78 and 150 of the 431 most reliable Ps-DHSs became bi-allelic in *Kdm6b*^*WT*^- and *Kdm4d*^*WT*^-injected zygotes, respectively, while their catalytic mutants had little effect (Fig. 2b, c, Extended Data Fig. 4i, and Table S5). This result indicates that both maternal H3K27me3 and H3K9me3 are involved in maternal allele inaccessibility. Importantly, Kdm6b-affected Ps-DHSs are largely devoid of oocyte DNA methylation, which is markedly different from Kdm4d-affected Ps-DHSs that locate at DNA hypermethylated regions (Fig. 2d). Consistently, Ps-DHSs specifically affected by Kdm6b, but not Kdm4d, overlap maternal allele-specific H3K27me3 (Extended Data Fig. 4j). These results suggest that maternal H3K27me3 and H3K9me3 restrict maternal allele accessibility at regions with hypomethylated and hypermethylated DNA, respectively.

## H3K27me3-dependent imprinting

**Extended data figure 5.**
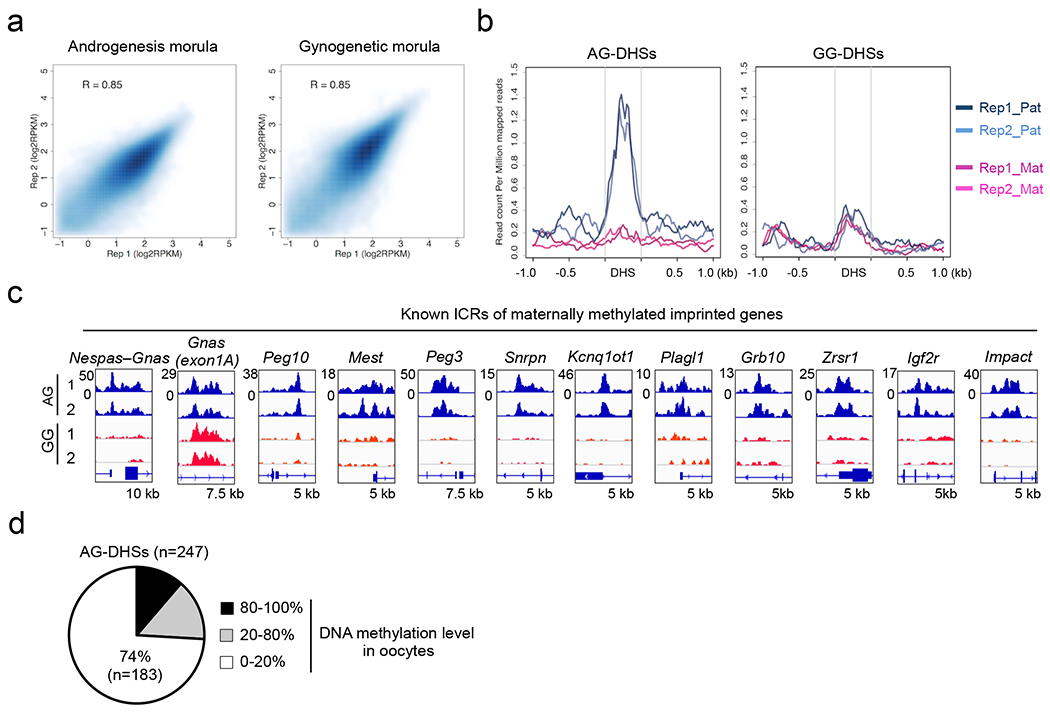
Androgenetic (AG)- and gynogenetic (GG)-specific DHSs in morula embryos, related to Figure 3 **a**, Scatter plot showing the correlation between biological duplicates of liDNase-seq for AG and GG morula embryos. **b**, Averaged SNP-tracked liDNase-seq signal intensity of paternal and maternal alleles in hybrid morula embryos. The data were obtained from morula embryos of a BDF1 and JF1 cross ^7^. Plots from the biological duplicates (*e.g.* BDF1_1 and BDF1_2) are shown. Note that paternal (JF1), but not maternal (BDF1), SNP reads are enriched in AG-DHSs (left panel), while neither SNP reads are enriched in GG-DHSs (right panel). **c**, Genome browser view of DHSs at known imprinting control regions (ICRs). The genomic locations of ICRs were defined previously ^9^. **d**, Pie chart showing AG-DHSs grouped based on their oocyte DNA methylation levels.

**Figure 3.**
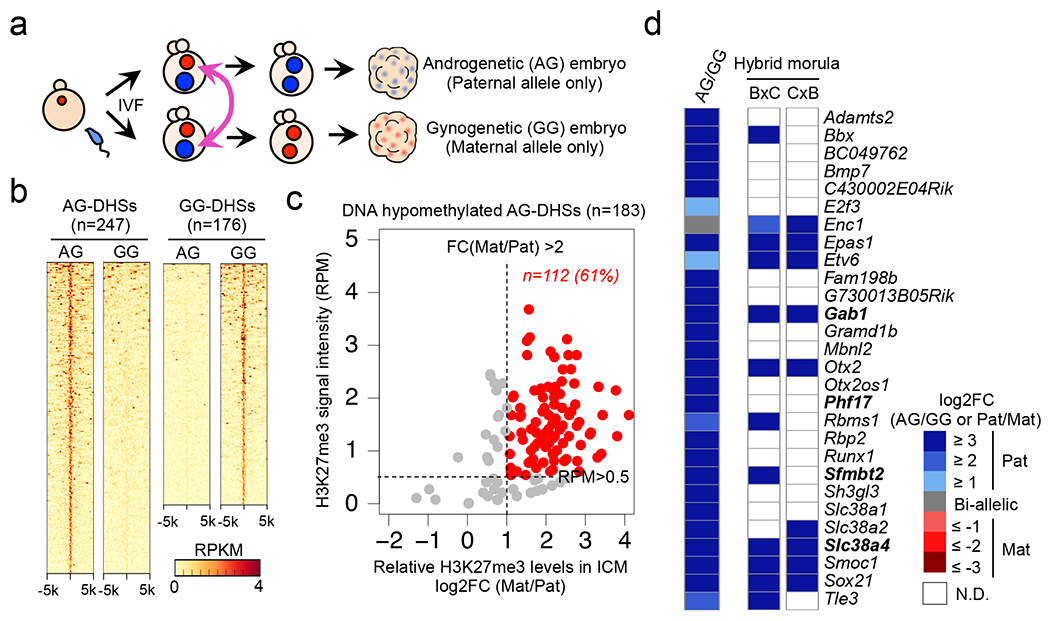
Genes with H3K27me3-marked AG-DHSs are paternally expressed in morula embryos **a**, Schematic for identifying parental allele-specific DHSs in morula embryos. **b**, Heat map showing AG-specific (AG-DHSs) and GG-specific DHSs (GG-DHSs) in morula embryos. Each row represents liDNase-seq signal intensity at a DHS ± 5 kb. **c**, Scatterplot showing allelic enrichment of H3K27me3 ChIP-seq signal at AG-DHSs ±1 kb in inner cell mass (ICM) of blastocyst embryos. AG-DHSs with [RPM>0.5, FC(Mat/Pat)>2] were considered to harbor maternal allele-biased H3K27me3 (red dots). **d**, Heat map showing parental allele-specific gene expression of putative H3K27me3-dependent imprinted genes. Genes expressed in AG morula embryos (RPKM>0.5) are shown. The left column represents the ratio of AG/GG gene expression. The two right columns represent relative gene expression in hybrid morula embryos. BxC; B6/CAST. CxB; CAST/B6. The 4 known non-canonical imprinted genes are indicated in bold. White boxes indicate ‘not determined (N.D.)’ due to lack of SNP reads (<20 reads).

To understand to what extent allelic DHSs exist at a later embryonic stage, we generated AG and GG morula embryos (Fig. 3a) and performed liDNase-seq (Extended Data Fig. 5a). Using the same criteria for allelic DHSs as in zygotes and excluding data of sex chromosomes, we identified 36,569, 247, and 176 of common DHSs, AG-specific DHSs (AG-DHSs), and GG-specific DHSs (GG-DHSs), respectively (Fig. 3b and Table S6). By SNP-tracking analyses of a public DHS profile of hybrid morula embryos ^7^, we confirmed that AG-DHSs, but not GG-DHSs, recapitulate the corresponding parental allele-specific DHSs (Extended Data Fig. 5b), indicating that AG-DHSs are physiological. Interestingly, AG-DHSs include almost all known maternally-methylated ICRs (Extended Data Fig. 5c). This finding raised the possibility that AG-DHSs could serve as indicators of genomic imprinting.

Because both gDMR and maternal H3K27me3 can contribute to maternal allele inaccessibility (Fig. 2), we determined their respective contribution to the generation of the 247 AG-DHSs. Analyses of the oocyte DNA methylome ^9^ identified 183 (74%) AG-DHSs in DNA hypomethylated regions (Extended Data Fig. 5d). Allelic H3K27me3 enrichment analysis revealed that 112 of the 183 are marked with maternal allele-biased H3K27me3 in inner cell mass (ICM) of blastocysts (Fig. 3c). Of the 112 AG-DHSs, 105 showed maternal allele-specific H3K27me3 enrichment in zygotes [RPM>0.5, FC(Mat/Pat)>4], suggesting that the maternal allele-biased H3K27me3 is inherited from zygotic maternal chromatin. By associating the 105 H3K27me3-marked AG-DHSs with their nearest genes, we obtained 76 genes (Table S7) as putative H3K27me3-dependent imprinted genes.

**Extended data figure 6.**
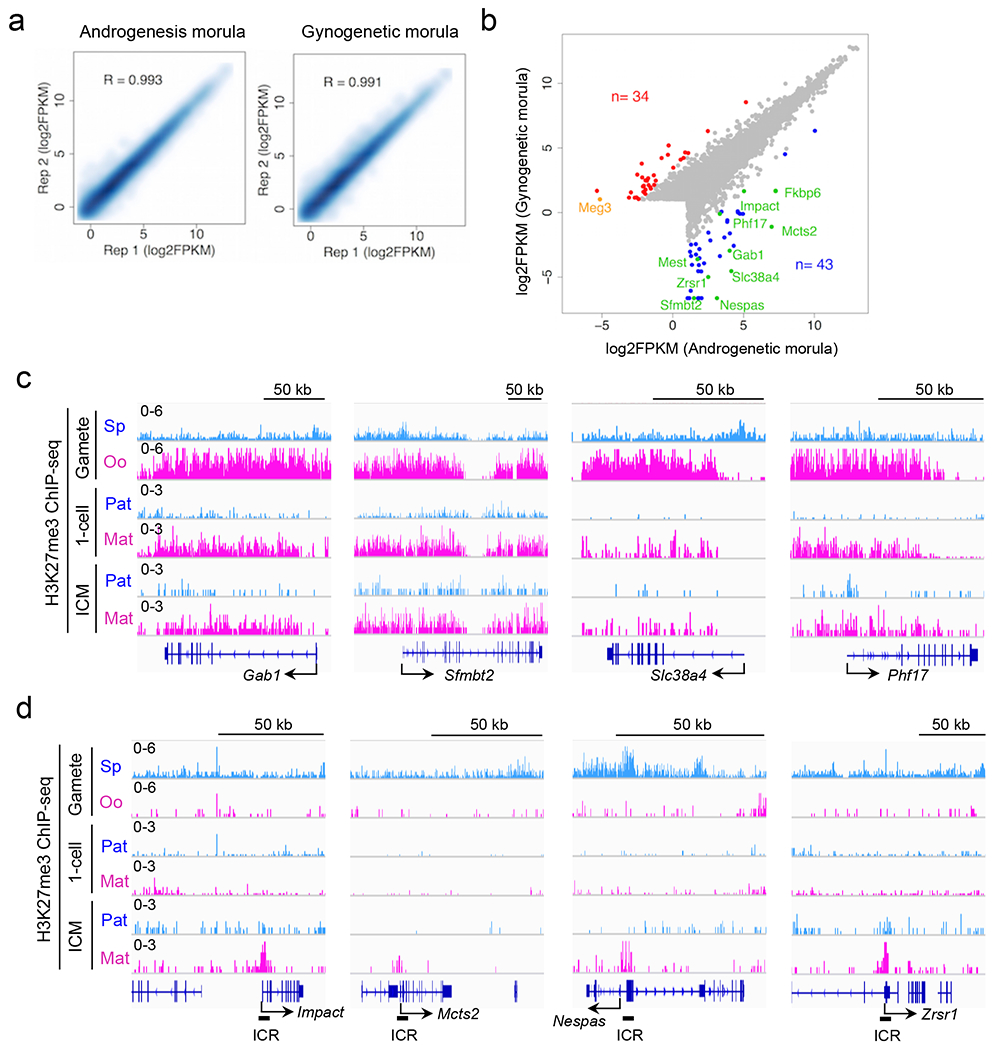
Allelic gene expression in morula embryos, related to Figure 3 **a**, Scatter plot showing the correlation between biological duplicates of RNA-seq samples. **b**, Scatterplot of gene expression levels in AG- and GG morula embryos. AG- and GG-specific differentially expressed genes (DEGs) (FC>10) are indicated in blue and red, respectively. Paternally- and maternally-expressed known imprinted genes are indicated in green and orange, respectively. **c**, Genome browser views of allelic H3K27me3 levels in non-canonical imprinted genes. Sp; sperm. Oo; MII-stage oocyte. ICM; inner cell mass of blastocysts. Paternal (Pat) and maternal (Mat) allele signals in 1-cell and ICM were based on SNP analyses. **d**, Genome browser views of allelic H3K27me3 levels in representative canonical imprinted genes. Known ICRs are indicated at the bottom of each canonical imprinted gene.

To determine if any of the 76 genes are indeed imprinted in preimplantation embryos, we performed RNA-seq analysis for AG and GG morula embryos (Extended Data Fig. 6a and Table S8). After confirming AG- or GG-specific expression of known imprinted genes (Extended Data Fig. 6b), we calculated the relative AG/GG expression levels for each candidate. Among the 76 genes, 28 were expressed in either AG or GG embryos (FPKM>0.5). Interestingly, 27 of the 28 genes exhibited biased (FC>2), and 23 genes exhibited highly biased (FC>8) expression in AG embryos (Fig. 3d, **left column**). Using a RNA-seq dataset of hybrid IVF morula embryos ^14^, we further confirmed that all 13 SNP-trackable genes exhibit paternal allele-specific expression (Fig. 3d, **right columns**). Importantly, these genes include *Sfmbt2*, *Gab1, Slc38a4*, and *Phf17* whose imprinted expression was suggested to be independent of oocyte DNA methylation^15–18^. These ‘non-canonical’ imprinted genes are coated with oocyte-specific H3K27me3 domains that are retained even in blastocysts (Extended Data Fig. 6c), which is in contrast to DNA methylation-dependent ‘canonical’ imprinted genes that are devoid of oocyte H3K27me3 (Extended Data Fig. 6d). Collectively, these results demonstrate that maternal H3K27me3 may serve as a DNA methylation-independent imprinting mark.

**Extended data figure 7.**
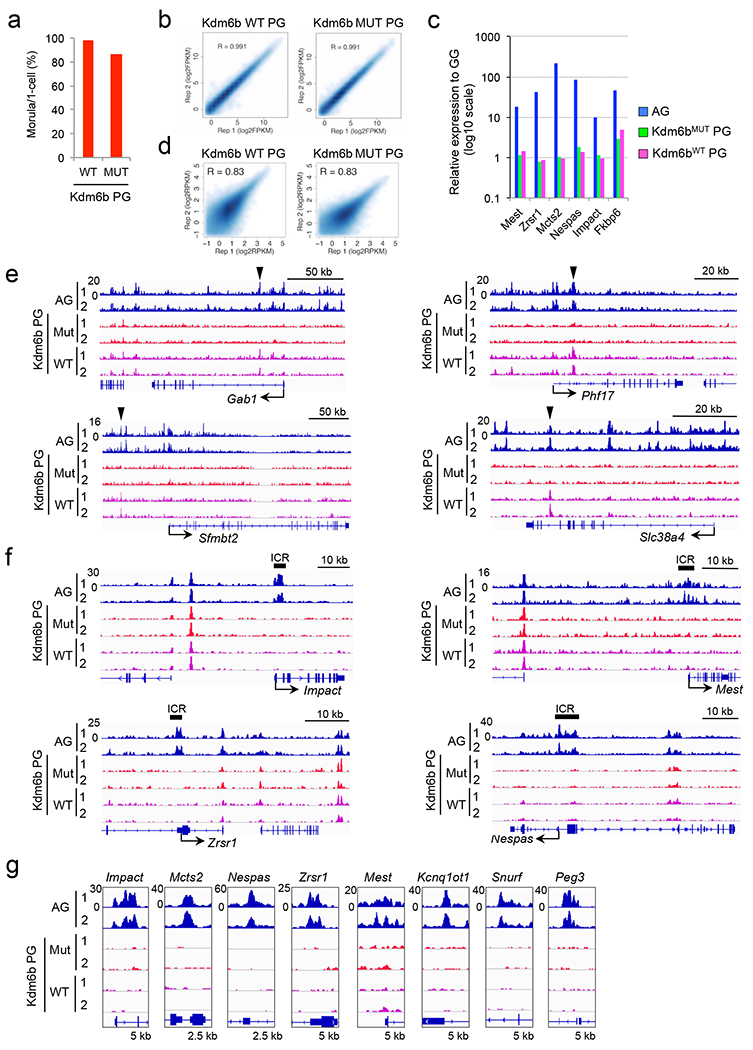
The effect of Kdm6b mRNA injection on maternal allele expression and accessibility, related to Figure 4 **a**, Developmental ratio of *Kdm6b*^*WT*^- and *Kdm6b*^*MUT*^-injected parthenogenetic (PG) embryos. The total embryo numbers examined were 60 (WT) and 58 (MUT). **b**, Scatter plot showing the correlation between biological duplicates of RNA-seq for *Kdm6b*^*WT*^- and *Kdm6b*^*MUT*^-injected PG embryos. **c**, Relative gene expression levels of canonical imprinted genes that are expressed in AG morula embryos (RPKM>0.5). Note that none are derepressed by Kdm6b^WT^ injection. **d**, Scatter plot showing the correlation between biological duplicates of liDNase-seq for *Kdm6b*^*WT*^- and *Kdm6b*^*MUT*^-injected PG embryos. **e, f**, Wide genome browser views of non-canonical (**e**) and canonical imprinted genes (**f**). The arrowheads indicate AG-DHSs at which chromatin accessibility is gained in *Kdm6b*^*WT*^-injected PG embryos (shown in figure 4e). Known imprinting control regions (ICRs) are indicated above each panel of canonical imprinted genes (**f**). **g**, Genome browser view of AG-DHSs of representative canonical imprinted genes.

**Figure 4.**
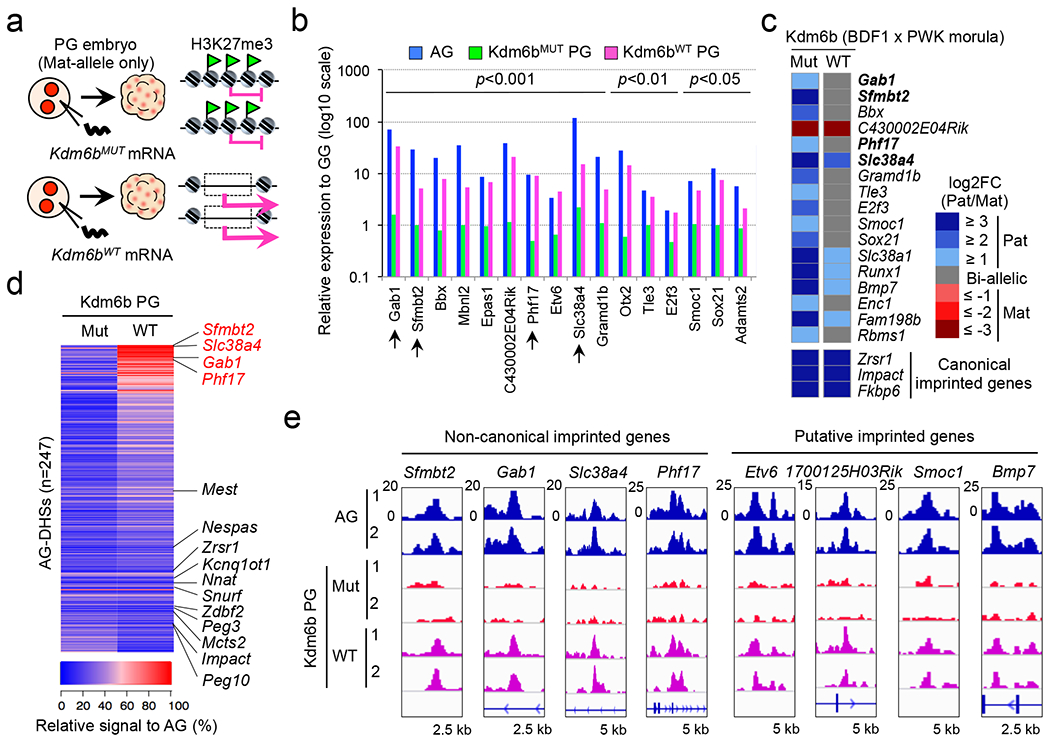
Maternal H3K27me3 serves as an imprinting mark **a**, Schematic for studying the role of H3K27me3 in maternal allele repression. *Kdm6b*^*MUT*^-injected parthenogenetic (PG) embryos were used as a negative control. **b,** Relative gene expression levels (log scale) of putative H3K27me3-dependent imprinted genes. Shown are genes expressed in AG morula embryos (RPKM>0.5) and significantly derepressed by Kdm6b^WT^. The expression level of gynogenetic (GG) morula embryos was set as 1. The genes are ordered by statistical significance (*p*-values by DEseq) between Kdm6b^WT^ and Kdm6b^MUT^ samples. Arrows indicate known non-canonical imprinted genes. **c**, Heat map showing parental allele-specific gene expression of putative H3K27me3-dependent imprinted genes in *Kdm6b*^*WT*^- and *Kdm6b*^*MUT*^-injected hybrid morula embryos. Among the 28 genes listed in figure 3d, those with >10 SNP reads in both samples are shown. Known non-canonical imprinted genes are indicated in bold. Allelic expression levels of representative canonical imprinted genes are shown at the bottom. **d**, Heat map showing the levels of chromatin accessibility at AG-DHSs in *Kdm6b*^*WT*^- and *Kdm6b*^*MUT*^-injected morula PG embryos. The DHS signal intensity in AG embryos was set as 100%. AG-DHSs are ordered by Δ(Kdm6b^WT^ – Kdm6b^MUT^). Known imprinted genes are indicated at right, with non-canonical imprinted genes shown in red. **e**, Genome browser view of gain-of-accessibility at AG-DHSs of putative H3K27me3-dependent imprinted genes.

To determine whether maternal H3K27me3 is responsible for maternal allele repression of the putative H3K27me3-dependent imprinted genes, we injected *Kdm6b*^*WT*^ or *Kdm6b*^*MUT*^ mRNAs into 1-cell stage parthenogenetic (PG) embryos (Fig. 4a). After verifying that the injection did not affect embryo development to the morula stage (Extended Data Fig. 7a), we performed RNA-seq analysis (Extended Data Fig. 7b). Of the 28 putative imprinted genes expressed in AG morula embryos (Fig. 3d), 16 were significantly derepressed in a catalytic activity-dependent manner, which include all 4 known non-canonical imprinted genes (Fig. 4b and Table S9). In contrast, canonical imprinted genes were not affected by *Kdm6b*^*WT*^ injection (Extended Data Fig. 7c), demonstrating that H3K27me3 is specifically required for maternal allele repression of the putative H3K27me3-dependent imprinted genes.

To demonstrate that Kdm6b-mediated maternal allele derepression occurs in a physiological context, we performed RNA-seq analysis in IVF-derived hybrid morula embryos that had been injected with *Kdm6b*^*WT*^ or *Kdm6b*^*MUT*^ mRNA at the 1-cell stage. Of the 28 putative imprinted genes, 17 had sufficient SNP reads, and 16 of them showed paternal allele-biased expression in *Kdm6b*^*MUT*^-injected embryos (Fig. 4c, Table S10). Notably, the extent of the paternal allelic bias of all these genes became milder in *Kdm6b*^*WT*^-injected embryos, while that of canonical imprinted genes was not affected (Fig. 4c). These data strongly suggest that imprinted expression of these genes depends on maternal H3K27me3.

To determine whether maternal allele derepression couples with gain of maternal chromatin accessibility, we performed liDNase-seq for *Kdm6b*^*WT*^- and *Kdm6b*^*MUT*^-injected PG morula embryos (Extended Data Fig. 7d). We found that Kdm6b^WT^, but not Kdm6b^MUT^, markedly increases chromatin accessibility in AG-DHSs of putative H3K27me3-dependent imprinted genes, including all 4 non-canonical imprinted genes (Fig. 4d, e and Extended Data Fig. 7e and Table S11). In contrast, ICRs of canonical imprinted genes were not affected (Fig. 4d and Extended Data Fig. 7f, g). These results suggest that maternal H3K27me3 restricts maternal allele accessibility to mediate H3K27me3-dependent genomic imprinting.

## Imprinting status in blastocysts

**Figure 5.**
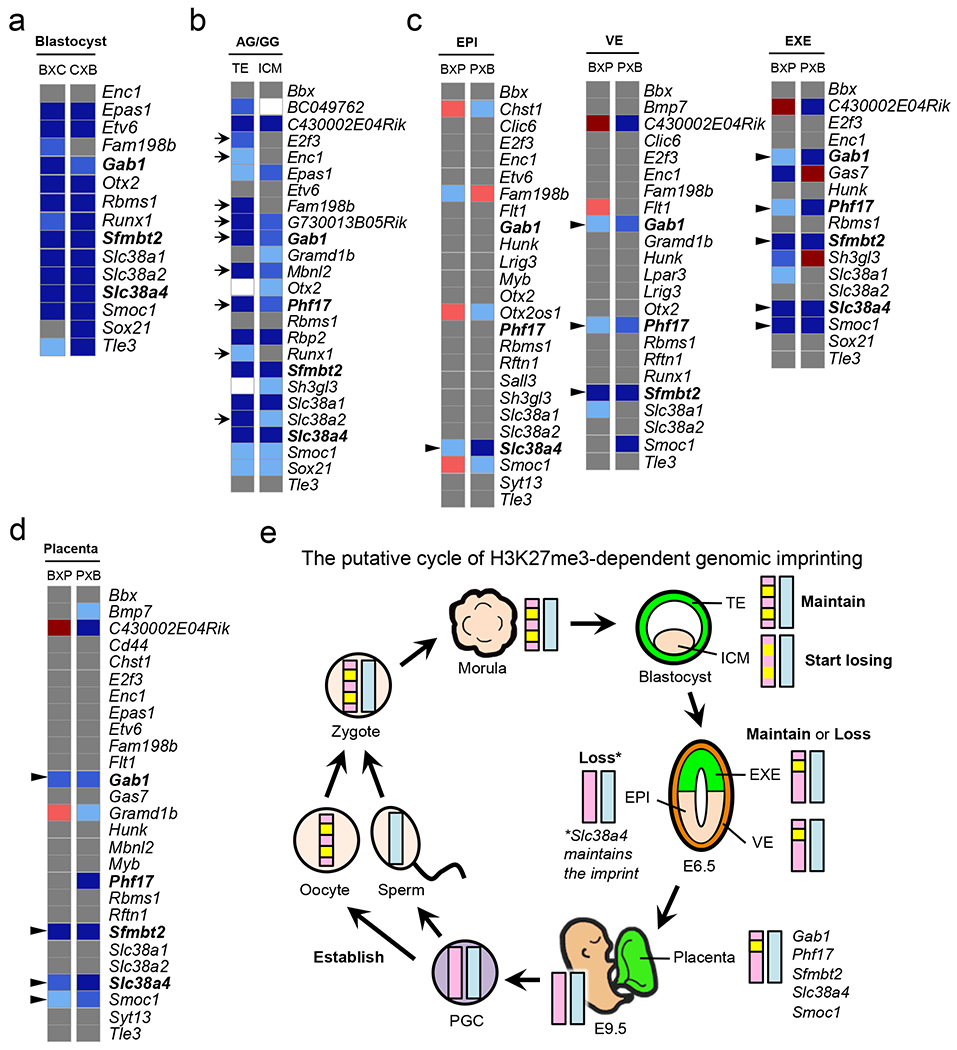
Cell lineage-specific dynamics of H3K27me3-dependent genomic imprinting **a**, Heat map showing parental allele-specific gene expression of putative H3K27me3-dependent imprinted genes in hybrid blastocyst embryos. BxC; B6/CAST. CxB; CAST/B6. Known non-canonical imprinted genes are indicated in bold in panels **a-d**. The color scheme in panels **a-d** follows figure 3d. **b**, Heat map showing androgenesis/gynogenesis (AG/GG) relative expression of putative H3K27me3-dependent imprinted genes in ICM and TE of blastocyst embryos. Arrows indicate genes showing a milder level of AG-bias in ICM when compared to TE. White boxes indicate ‘not determined’ due to low gene expression levels (RPKM<0.5). **c**, Heat map showing parental allele-specific gene expression of putative H3K27me3-dependent imprinted genes in epiblast (EPI), visceral endoderm (VE), and extra-embryonic ectoderm (EXE) of E6.5 embryos. Genes with >20 SNP reads in both reciprocal crosses are shown. BxP; B6/PWK. PxB; PWK/B6. Arrowheads indicate genes showing imprinted expression. **d**, Heat map showing parental allele-specific gene expression of putative H3K27me3-dependent imprinted genes in pure fetus-derived E9.5 placenta cells. Genes with >20 SNP reads in both reciprocal crosses are shown. Arrowheads genes showing imprinted expression. **e**, Model illustrating the fate of H3K27me3-dependent genomic imprinting during development.

We next analyzed the imprinting status of putative H3K27me3-dependent imprinted genes in blastocyst embryos by SNP tracking of recently published datasets ^14^. Of the 28 genes imprinted in morula embryos (Fig. 3d), 15 had sufficient SNP reads in both reciprocal crosses (Fig. 5a). Among them, 12 (80%) showed paternal allelic expression in both crosses (Fig. 5a), demonstrating that H3K27me3-dependent imprinting is largely maintained in blastocysts.

**Extended data figure 8.**
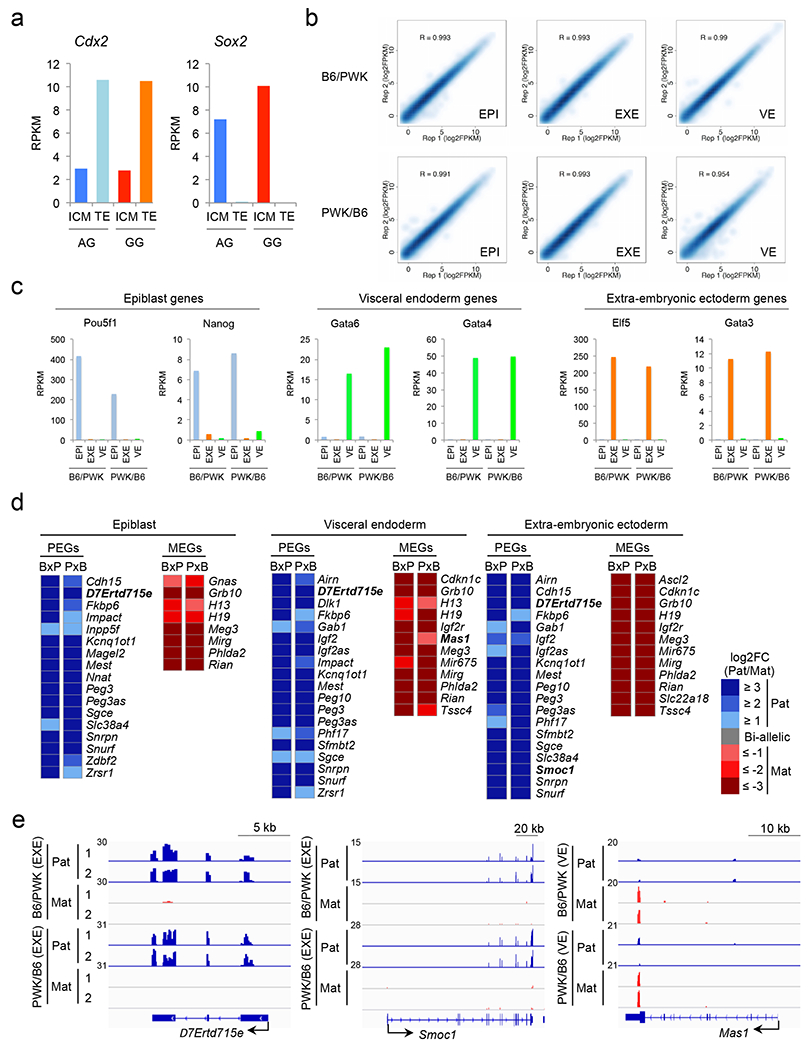
Genomic imprinting in E6.5 embryos, related to Figure 5 **a**, Expression levels of marker genes for TE (*Cdx2*) and ICM (*Sox2*) in the samples. **b**, Scatter plot showing the correlation between biological duplicates of the E6.5 epiblast (EPI), visceral endoderm (VE), and extra-embryonic ectoderm (EXE) RNA-seq samples from both B6xPWK and PWKxB6 crosses. **c**, Bar graphs showing the expression levels of marker genes for epiblast (*Pou5f1* and *Nanog*), extra-embryonic ectoderm (*Elf5* and *Gata3*), and visceral endoderm genes (*Gata6* and *Gata4*) in the samples. **d**, Heat map showing paternally-expressed genes (PEGs) and maternally-expressed genes (MEGs) in epiblast, visceral endoderm, and extra-embryonic ectoderm of E6.5 embryos. BxP; B6/PWK. PxB; PWK/B6. All genes showing parental allele-specific expression (FC>2 in both BxP and PxB) in each sample are shown. Genes not previously known to be imprinted are indicated in bold. **e**, Genome browser view of RNA-seq data of newly identified imprinted genes. *D7Ertd715e* and *Smoc1* are paternally expressed, and *Mas1* is maternally expressed. EXE, extra-embryonic ectoderm. VE, visceral endoderm.

Since previous studies have indicated that *Gab1*, *Sfmbt2*, and *Phf17* are imprinted only in extra-embryonic tissues ^19–21^, we examined their imprinting status in ICM. We isolated TE and ICM cells from AG and GG blastocysts and performed RNA-seq analysis (Table S12). Marker gene expression confirmed no cross-contamination (Extended Data Fig. 8a). Of the 28 putative imprinted genes (Fig. 3d), 23 and 24 are expressed in TE and ICM, respectively (RPKM>0.5). Of these, 18 (78%) in TE and 16 (67%) in ICM show AG-biased expression (FC>2) (Fig. 5b). Notably, 9 genes show weaker AG-bias in ICM compared to TE (Fig. 5b, **arrows**), suggesting that H3K27me3-dependent imprinting might start to diminish in ICM.

## Post-implantation imprinting dynamics

To determine the imprinting status in post-implantation embryos, we dissected hybrid E6.5 embryos into epiblast (EPI), visceral endoderm (VE), and extra-embryonic ectoderm (EXE), and performed RNA-seq analysis (Extended Data Fig. 8b and Table S13). We confirmed their cell identify by analyzing cell lineage-specific marker gene expression ^22^ (Extended Data Fig. 8c) and identified 17 paternally-expressed genes (PEGs) and 8 maternally-expressed genes (MEGs) in EPI, 19 PEGs and 12 MEGs in both VE and EXE, which include new imprinted genes, such as *D7Ertd715e* (also known as *Snhg14*), *Smoc1*, and *Mas1* (Extended Data Fig. 8d, e and Table S13).

Among the 76 putative H3K27me3-dependent imprinted genes (Table S7), 25, 23, and 17 genes had enough SNP reads in both reciprocal crosses in EPI, VE, and EXE, respectively (Fig. 5c). We found that 1, 3, and 5 genes are paternally expressed in EPI, VE, and EXE, respectively (Fig. 5c, **arrowheads**). The genes imprinted in EXE include the 4 non-canonical imprinted genes, *Gab1*, *Phf17*, *Sfmbt2*, and *Slc38a4*, and a new imprinted gene, *Smoc1* (Fig. 5c). These results suggest that H3K27me3-dependent imprinting is completely erased in the epiblast with the exception of *Slc38a4*, but some are maintained in the extra-embryonic cell lineages.

**Extended data figure 9.**
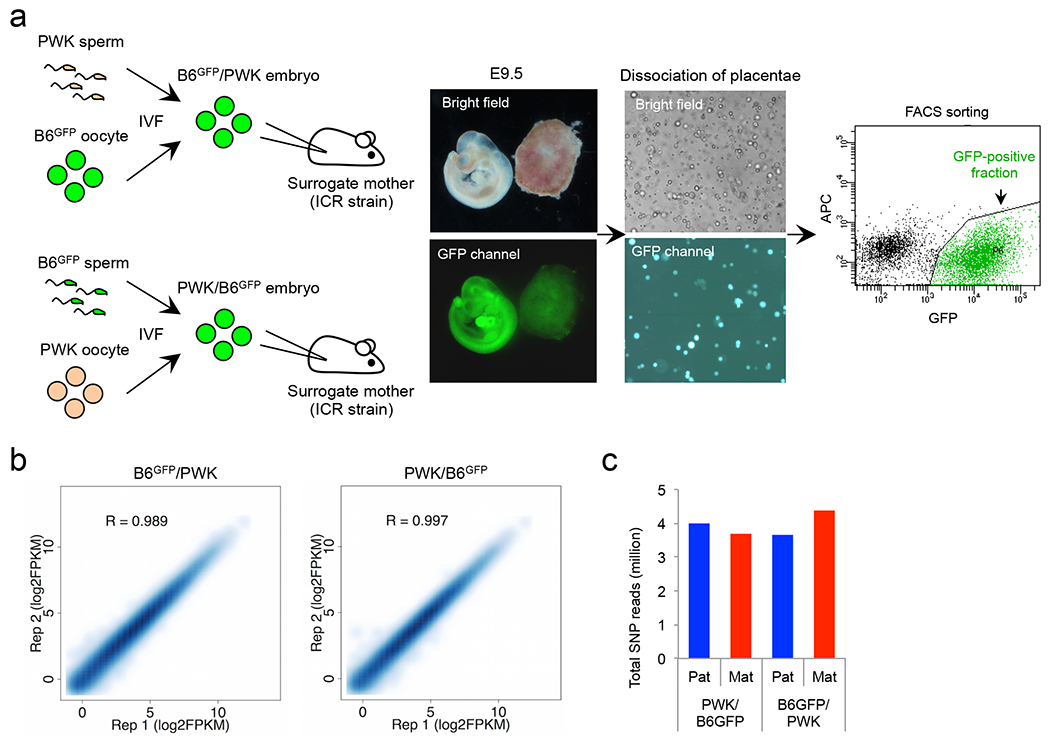
Sample preparation and quality verification, related to Figure 5 **a**, Experimental scheme of placenta cell purification. Sperm or oocytes were collected from B6^GFP^ mice, and *in vitro* fertilized with the counterparts collected from the PWK strain. Embryos were transplanted into surrogate mothers. The placentae were harvested at E9.5, and dissociated into single cells by trypsin treatment before FACS sorting of GFP-positive cells. **b**, Scatter plot showing the correlation between biological duplicates of RNA-seq samples. **c**, Total numbers of the paternal and maternal SNP reads in the purified placental cells.

**Extended data figure 10.**
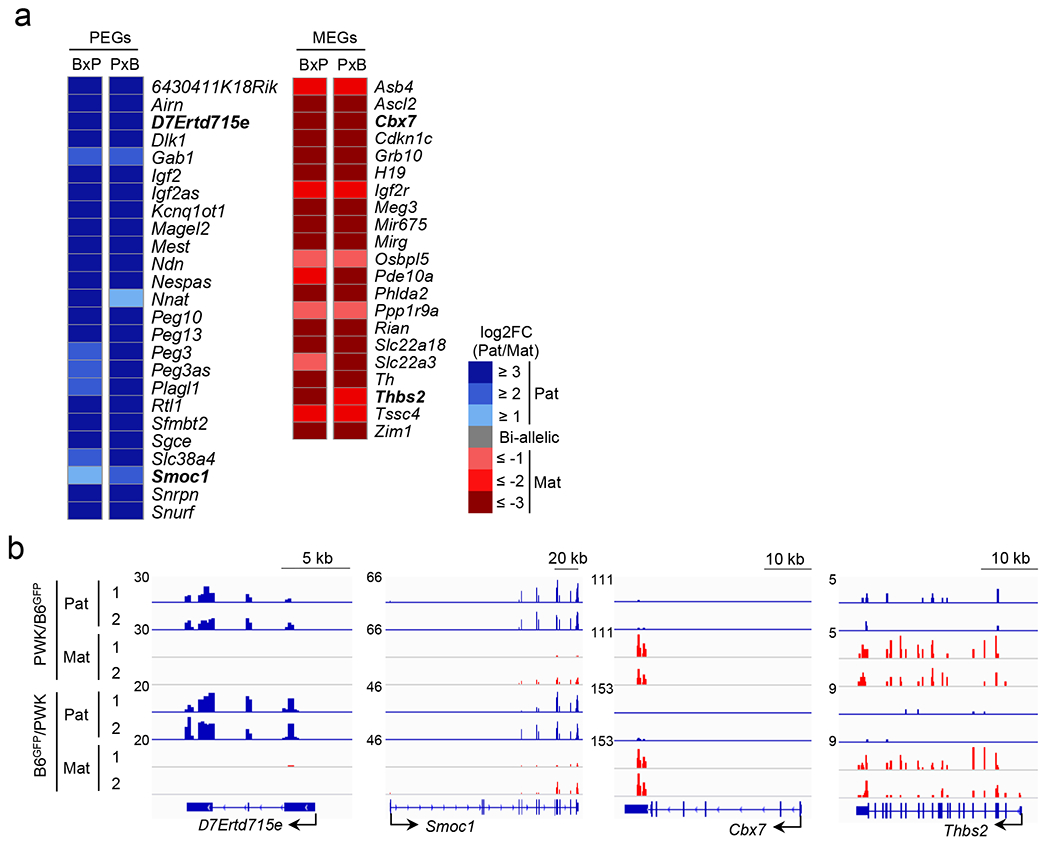
Genomic imprinting in E9.5 placentae, related to Figure 5 **a**, Heat map showing paternally-expressed genes (PEGs) and maternally-expressed genes (MEGs) in E9.5 placentae. BxP; B6/PWK. PxB; PWK/B6. All genes exhibiting parental allele-specific expression (FC>2 in both BxP and PxB) are shown. Genes not previously known to be imprinted are indicated in bold. **b**, Genome browser view of RNA-seq data of newly identified imprinted genes. *D7Ertd715e* and *Smoc1* are paternally expressed, and *Cbx7* and *Thbs2* are maternally expressed.

To analyze the imprinting status in E9.5 placentae avoiding possible maternal cell contamination, we purified fetus-derived placental cells from GFP transgenic embryos by FACS-sorting (Extended Data Fig. 9a) and performed RNA-seq analysis (Extended Data Fig. 9b and Table S14). After confirming cell purity by demonstrating comparable total SNP reads from parental alleles (Extended Data Fig. 9c), we identified 25 PEGs and 21 MEGs, which include new imprinted genes, such as *D7Ertd715e, Smoc1, Cbx7* and *Thbs2* (Extended Data Fig. 10a, b, and Table S14). Among the 76 putative H3K27me3-dependent imprinted genes, 27 genes had sufficient SNP reads in both reciprocal crosses (Fig. 5d). Among them, *Gab1*, *Sfmbt2*, *Slc38a4*, and *Smoc1* are paternally expressed (Fig. 5d). Imprinting of *Phf17* in one cross was weak (FC=1.87) (Fig. 5d and Table S14), which was consistent with a previous study ^23^. Taken together, our data not only identify *Smoc1* as a new H3K27me3-dependent imprinted gene, but also suggest that most H3K27me3-dependent imprinted genes are transiently imprinted in preimplantation embryos, while some remain imprinted in the extra-embryonic cell lineage (Fig. 5e).

## Discussion

Since the identification of DNA methylation as a genomic imprinting mark more than 20 years ago ^24–26^, it has been the only known mammalian germline imprinting mark ^4^. However, recent studies have identified several imprinted genes capable of maintaining paternal allele-specific expression in the absence of oocyte DNA methylation ^21,27^, suggesting the existence of a DNA methylation-independent imprinting mechanism. Here we revealed that these non-canonical imprinted genes harbor high level of oocyte-specific H3K27me3, and that loss of H3K27me3 results in loss-of-imprinting. Although previous studies have revealed a link between a repressed allele and repressive histone modifications, including H3K27me3, at certain imprinted loci ^28–37^, the imprinting status of these loci originally depends on gDMRs ^17,29,37,38^. Consistently, ectopic removal of H3K27me3 specifically affected non-canonical imprinted genes, indicating that the regulatory mechanism of H3K27me3-dependent imprinting is fundamentally different from that of gDMR-mediated canonical imprinting.

The dynamics of H3K27me3-dependent imprinting is strikingly different from DNA methylation-dependent imprinting which is largely maintained in both embryonic and extra-embryonic lineages ^39^. The H3K27me3 imprint mark is likely established during oogenesis and maintained in preimplantation embryos (Fig. 5e). While it begins to dilute in ICM and is almost completely lost in the epiblast of E6.5 embryos, it is maintained in some genes at least until E9.5 placenta. Further investigation is warranted to understand why and how these genes are selected to maintain imprinting and why they use H3K27me3, instead of DNA methylation, as an imprinting mark, as well as how cell lineage-specific imprinting is achieved. Furthermore, what other organisms may conserve H3K27me3-dependent genomic imprinting is a fascinating question given that flowering plants also adopt this mechanism ^40,41^.

## METHODS

### Isolation of maternal and paternal pronuclei from PN5 stage zygotes

All animal studies were performed in accordance with guidelines of the Institutional Animal Care and Use Committee at Harvard Medical School. MII-stage oocytes were collected from 8 week-old B6D2F1/J (BDF1) females superovulated by injecting 7.5 I.U. of PMSG (Millipore) and hCG (Millipore). For *in vitro* fertilization (IVF), MII oocytes were inseminated with activated spermatozoa obtained from the caudal epididymis of adult BDF1 male mice in HTF medium supplemented with 10 mg/ml bovine serum albumin (BSA; Sigma-Aldrich). Spermatozoa capacitation was attained by 1 h incubation in the HTF medium. Zygotes were cultured in a humidified atmosphere with 5% CO_2_/95% air at 37.8°C. At 10 hours post-fertilization (hpf), zygotes were transferred into M2 media containing 10 μg/ml cytochalasin B (Sigma-Aldrich). Zona pellucidae were cut by a Piezo impact-driven micromanipulator (Prime Tech Ltd., Ibaraki, Japan) and the pronuclei were isolated from the zygotes. At 12 hpf (PN5-stage), isolated pronuclei were washed with 0.2% BSA/PBS, transferred into Eppendorf LoBind 1.5 ml tubes, and placed on ice until DNase I treatment. For each experiment, 150–200 pronuclei were collected and prepared for liDNase-seq. The parental pronuclei were distinguished by (1) the distance from the second polar body and (2) the size of the pronucleus.

### Preparation of androgenetic (AG) and gynogenetic (GG) embryos

MII oocytes were collected from 8 week-old superovulated BDF1 females and inseminated with BDF1 sperm. At 7 hpf, zygotes were transferred into M2 media containing 5 μg/ml cytochalasin B, and parental pronuclei were exchanged by using a Piezo impact-driven micromanipulator. The sendai virus (HVJ, Cosmo-bio) was used for fusing karyoplasts with cytoplasms as previously described ^42^. After reconstruction, embryos were cultured in KSOM.

When collecting embryos for RNA-seq or/and liDNase-seq, we removed zona pellucida (ZP) by a brief exposure to Acid tyrode’s solution (Sigma-Aldrich), then the embryos were washed with M2 media, and then 0.2% BSA/PBS. For liDNase-seq, 10 morula embryos were transferred into an Eppendorf LoBind 1.5 ml tube, and placed on ice until DNase I treatment. For RNA-seq, seven to ten embryos were transferred into a thin-walled RNase-free PCR tubes (Ambion). The 2-cell and morula embryos were collected at 30 and 78 hpf, respectively. When preparing α-amanitin treated 2-cell embryos, 5 hpf zygotes were transferred into KSOM containing 25 μg/ml α-amanitin (Sigma-Aldrich) and cultured in the presence of α-amanitin until collection (30 hpf).

ICM and TE were isolated as described previously ^43^ with slight modifications. Briefly, AG and GG embryos at 120 hpi were treated with Acid tyrode’s solution to remove ZP. After being washed in M2 media, the embryos were incubated in KSOM containing rabbit anti-mouse lymphocyte serum (Cedarlane, 1:8 dilution) for 45 min at 37°C. After being washed in M2 media, they were transferred into KSOM containing guinea pig complement (MP Biomedicals, 1:3.3 dilution). After incubation for 30 min at 37°C, lysed TE cells were removed by pipetting with a glass capillary. The remaining ICM clumps were incubated in 0.25% Trypsin/EDTA (Thermo Fisher, 25200) for 10 min at 37°C, and then dissociated into single cells to avoid contamination of lysed TE cells. 100-200 cells were collected for RNA-seq.

### Isolation of GV nuclei from fully-grown oocytes

Fully-grown GV-stage oocytes were obtained from 3-week-old BDF1 mice 44-48 h after injection with 5 I.U. PMSG. The ovaries were transferred to M2 media. The ovarian follicles were punctured with a 30-gauge needle, and the cumulus cells were gently removed from the cumulus–oocyte complexes using a narrow-bore glass pipette. The oocytes were then transferred into α-MEM (Life technologies, 12571-063) supplemented with 5% Fetal Bovine Serum (FBS) (Sigma-Aldrich, F0926), 10 ng/ml Epidermal Growth Factor (Sigma-Aldrich, E4127), and 0.2 mM 3-isobutyl-1-methylxanthine (IBMX; Sigma–Aldrich). One hour after collection, GV oocytes exhibiting visible perivitelline spaces, which have the surrounding-nucleolus (SN)-type chromatin, were culled ^44^. They were then incubated in M2 media containing 10 μg/ml cytochalasin B, 0.1 μg/ml colcemid (Sigma-Aldrich), and 0.2 mM IBMX for 15 min. Then, GV nuclei were isolated by using a Piezo-driven micromanipulator. After washing with 0.2% BSA/PBS, the GV nuclei were transferred into an Eppendorf LoBind 1.5 ml tube. For each experiment, 115-150 GV nuclei were collected for liDNase-seq.

### Dissection of E6.5 embryos and FACS sorting of GFP-positive E9.5 placental cells

To obtain C57BL6(B6)/PWK hybrid embryos, we used a natural mating scheme. To obtain PWK/B6 hybrid embryos, we used *in vitro* fertilization of PWK oocytes with B6 sperm, and the 2-cell embryos were transferred into surrogate ICR strain mothers. Dissection of E6.5 embryos into EPI, EXE, and VE was performed as described previously ^45^. To collect E9.5 placental cells, we purchased the B6^GFP^ mice from Jackson laboratory [C57BL/6-Tg(CAG-EGFP)131Osb/LeySopJ, Stock number 006567]. MII oocytes and sperms were collected from superovulated 8-week old B6^GFP^ or PWK mice. After *in vitro* fertilization, the 2-cell embryos were transferred into surrogate ICR strain mothers. At E9.5, placentae were harvested, cut into ~0.5 mm pieces, transferred into 50 ml tubes, and treated with 2 ml of 0.25% Trypsin-EDTA (Thermo Fisher Scientific, 25200) at 30°C for 15 min in a shaker at 200 rpm to dissociate placental cells. Trypsin treatment was stopped by the addition of 2 ml DMEM containing 10% FBS. After pipetting, the tubes were centrifuged and the pelleted cells were washed with 0.2%BSA/PBS three times. DAPI was added at the final concentration of 1 μM in the final cell suspension. The GFP-positive cells were sorted using a BD FACSaria machine (BD Biosciences) with DAPI positive cells excluded as dead cells. Approximately 10,000-20,000 GFP-positive cells were collected from each placenta, which corresponded to 40-60% of total placental cells.

### Plasmid construction and mRNA preparation

To generate the *Kdm6b*^*WT*^ construct, the cDNA encoding the carboxyl-terminal part containing the catalytic domain (amino acid 1025-End) was amplified ^12^. The PCR amplicon was cloned between a Flag tag and poly(A) of the pcDNA3.1-Flag-poly(A)83 plasmid ^46^. The H1390A *Kdm6b*^*MUT*^ construct ^47^ were generated by using PrimeSTAR mutagenesis (TAKARA). Primers used for the mutagenesis are 5′-CCAGGCgctCAAGAGAATAACAATTTCTGCTCAGTCAACATCAAC-3′ and 5’-CTCTTGagcGCCTGGCGTTCGGCTGCCAGGGACCTTCATG-3’. All constructs were verified by DNA sequencing. The plasmids for wild-type and H189A mutant *Kdm4d* were previously described ^13^.

After linearization by a restriction enzyme, the construct was purified with phenol-chloroform extraction. mRNA was synthesized by *in vitro* transcription using a mMESSAGE mMACHINE T7 Ultra Kit (Life technologies) according to manufacturer’s instructions. The synthesized mRNA was purified by lithium chloride precipitation and diluted with nuclease-free water. mRNA aliquots were stored in −80°C until use.

### mRNA injection

MII oocytes were collected from superovulated 8 week-old BDF1 females and inseminated with BDF1 sperm. At 2.5 hpf, fertilized oocytes were transferred into M2 media and mRNA was injected using a Piezo impact-driven micromanipulator. mRNA injection was completed by 4 hpf. The mRNA concentrations of *Kdm6b*^*WT*^ and *Kdm6b*^*MUT*^ were 1.8 μg/μl, and those of *Kdm4d*^*WT*^ and *Kdm4d*^*MUT*^ were 1.5 μg/μl. When preparing *Kdm6b*-injected PG embryos, MII oocytes were chemically activated by treating with 3 mM SrCl_2_ in Ca^2+^-free KSOM containing 5 μg/ml cytochalasin B. At 4 hrs post-activation (hpa), the embryos were washed with KSOM. At 5 hpa, they were injected with mRNA.

### Whole mount immunostaining

Zygotes were fixed in 3.7% paraformaldehyde (PFA) in PBS containing 0.2% Triton for 20 min. After 4x washes with PBS containing 10 mg/ml BSA (PBS/BSA), zygotes were treated with primary antibodies at 4°C overnight. The primary antibodies used in this study were mouse-anti-H3K27me3 (1/500, Active Motif, 61017), rabbit anti-H3K9me3 (1/500, Millipore, 07-442), and rabbit anti-FLAG (1/2000, Sigma-Aldrich, F7524). After 3x washes with PBS/BSA, samples were incubated with a 1:250 dilution of fluorescein isothiocyanate–conjugated anti-mouse IgG (Jackson Immuno-Research) or Alexa Flour 568 donkey anti-rabbit IgG (Life technologies) for 1 h. The zygotes were then mounted on a glass slide in Vectashield anti-bleaching solution with 4’,6-diamidino-2-phenylindole (DAPI) (Vector Laboratories, Burlingame, CA). Fluorescence was detected under a laser-scanning confocal microscope with a spinning disk (CSU-10, Yokogawa) and an EM-CCD camera (ImagEM, Hamamatsu) or Zeiss LSM800.

All images were acquired and analyzed using the Axiovision software (Carl Zeiss). The fluorescent signal intensity was quantified with the Axiovision software. Briefly, the signal intensity within the maternal pronuclei was determined, and the cytoplasmic signal was subtracted as background. Then, the averaged signal intensity of the no-injection control zygotes was set as 1.0.

### Low-input DNase-seq

Low-input DNase-seq libraries were prepared as previously described with minor modifications ^7^. Embryos or nuclei collected in 1.5 ml tubes were resuspended in 36 μl lysis buffer (10 mM Tris-HCl, pH 7.5, 10 mM NaCl, 3 mM MgCl2, 0.1% Triton X-100) and incubated on ice for 5 min. DNase I (10 U/μl, Roche) was added to the final concentration of 80 U/ml (for the GV nucleus sample) or 40 U/ml (for all the other samples) and incubated at 37 °C for exactly 5 min. The reaction was stopped by adding 80 μl Stop Buffer (10 mM Tris-HCl, pH 7.5, 10 mM NaCl, 0.15% SDS, 10 mM EDTA) containing 2 μl Proteinase K (20 mg/ml, Life technologies). Then 20 ng of a circular carrier DNA [a pure plasmid DNA without any mammalian genes purified with 0.5x Beckman SPRIselect beads (Beckman Coulter) to remove small DNA fragments] was added. The mixture was incubated at 50 °C for 1 hr, then DNA was purified by extraction with phenol-chloroform and precipitated by ethanol in the presence of linear acrylamide (Life technologies) overnight at −20 °C. Precipitated DNA was resuspended in 50 μl TE (2.5 mM Tris, pH 7.6, 0.05 mM EDTA), and the entire volume was used for sequencing library construction.

Sequencing library was prepared using NEBNext Ultra II DNA Library Prep Kit for Illumina (New England Biolabs) according to the manufactures’ instruction with the exception that the adaptor ligation was performed with 0.03 μM adaptor in the ligation reaction for 30 minutes at 20 °C and that PCR amplification was performed using Kapa Hifi hotstart readymix (Kapa Biosystems) for 8-cycles. The PCR products were purified with x1.3 volume of SPRIselect beads (Beckman Coulter) and then size selected with x0.65 volume followed by x0.7 volume of SPRIselect beads. The sample was eluted in 24 μl TE. The number of cycles needed for the second PCR amplification was determined by qPCR using 1 μl of the 1:1,000 diluted samples. The remaining 23 μl of the samples was then amplified with Kapa Hifi hotstart readymix (we used 7 cycles for all samples in this study). The PCR product was purified with x1.3 volume of SPRIselect beads and then size selected with x0.65 volume followed by x0.7 volume of SPRIselect beads. The DNA was eluted in 30 μl of TE and quantified by Qubit dsDNA HS assay kit (Thermo Fisher Scientific, Q32854) and Agilent high sensitivity assay kit (Agilent Technologies). The libraries were sequenced on a Hiseq2500 with single-end 100 bp reads (Illumina).

### RNA-sequencing

RNA-seq libraries were prepared as previously described ^13^. Briefly, reverse transcription and cDNA amplification were performed using whole embryo lysates with SMARTer Ultra Low Input RNA cDNA preparation kit (Clontech, 634890). When processing 2-cell AG, GG and α-amanitin-treated IVF embryo samples, 1 μl of 1:40,000 diluted ERCC (External RNA Controls Consortium) standard RNA (Life technologies) was added to each of the tubes at the step of cell lysis. cDNAs were then fragmented using the Covaris M220 sonicator (Covaris) with microTUBE-50 (Covaris) into average 150-160 bp fragments. The fragmented cDNAs were end-repaired, adaptor ligated and amplified using NEBNext Ultra DNA Library Prep Kit for Illumina according to the manufacturer’s instruction (New England Biolabs). Single end 100 bp sequencing was performed on a HiSeq2500 sequencer (Illumina).

### liDNase-seq data analysis

Reads of liDNase-seq data were firstly trimmed of low quality and adapter with trim_galore, and then mapped to the mouse genome (mm9) using Bowtie v0.12.9. ‘-m 1’ parameter to keep unique mapping hits. The reads with mapping quality (MAPQ) ≤ 10 or redundant reads that mapped to the same location with the same orientation were removed with SAMtools^48^. The DHS peaks in liDNase-seq data were identified by Hotspot program with FDR <= 0.01 ^49^. The DHS peaks from all 33 libraries were merged using ‘bedtools merge’ from bedtools ^50^. The number of reads in each DHS for each library was calculated using ‘multiBamSummary’ from deepTools ^51^ and normalized to the total number of mapped reads and to the length of DHS (possibility of a tag located on a position per 1 kb per million mapped reads). Reads of sex chromosomes were removed because the number of sex chromosomes is different between the parental pronuclei and between androgenetic and gynogenetic embryos. The Pearson correlation coefficient (r) of tag densities at genome-wide DHSs was calculated to measure the correlation between replicates. For identification of parental allele-specific DHSs in zygotes and morula embryos, we used a stringent cutoff (RPKM mean>2, RPKM>1 in all replicates in a biased allele, and mean value fold change larger than 4 between the two alleles). The 431 most reliable Ps-DHSs were identified by applying an additional criterion ‘RPKM>1 in all replicates of paternal PNs of microinjected zygotes’ to Ps-DHSs. The RefSeq gene assembly (mm9) from the UCSC Genome Browser database and CGIs previously defined ^9^ were used as genomic feature distribution analysis in Extended Data Fig. 1d and 1e.

### RNA-seq data analysis

We constructed a custom reference sequence combining mouse genome (mm9) with the ERCC control. Reads of RNA-seq were mapped to the reference genome with TopHat v2.0.6 ^52^ or STAR (https://github.com/alexdobin/STAR). All programs were run with default parameters unless otherwise specified. Uniquely mapped reads were subsequently assembled into transcripts guided by the reference annotation (UCSC gene models) with featureCounts from subread-v1.5.1^53^. For all 2-cell RNA-seq libraries, library size factors were estimated with ‘estimateSizeFactors’ function form R package DESeq ^54^ only using ERCC read counts. After the library size was normalized, the expression level of each gene was quantified with normalized FPKM (fragments per kilobase of exon per million mapped fragments). The Pearson correlation coefficient (r) of gene expression level was calculated to indicate the correlation between duplicates. For identification of newly synthesized transcripts at the 2-cell stage, we firstly filtered out statistically non-significant genes between AG or GG and α-amanitin treated 2-cell embryo. To this end, adjusted P value was calculated with ‘nbinomTest’ function form R pakage DESeq using a negative binomial model, and only genes with FDR<0.05 were selected. We then applied additional cutoffs [Mean FPKM (AG or GG)>2 and fold-change (FC) (AG/Ama or GG/Ama)>2]. As a result, 4,381 and 3,916 genes were identified as newly synthesized genes in AG and GG 2-cell embryos, respectively. For identifying AG- and GG-specific DEGs in 2-cell embryos, the gene expression level (FPKM) of each gene in α-amanitin 2-cell embryos was subtracted from that of AG and GG embryos. Genes showing FC (AG/GG or GG/AG)>10 were identified as DEGs.

### WGBS and H3K27me3 ChIP-seq data analyses

The DNA methylation level at DHSs was calculated using methpipe v3.4.2 ^55^. When calculating the DNA methylation level at each DHS, to get enough coverage of WGBS reads, we extended each DHS to both up and downstream 2 kb to include more nearby CpG sites. The oocyte-methylated gDMR was defined by >80% methylation in oocytes and <20% in sperm ^9^. For Extended Data Fig. 4a, “bedtools makewindows” were used to generate a set of non-overlapped 1 kb bins for the ±100 kb flanking region of Ps-DHSs. For H3K27me3 ChIP-seq analysis, Bed files were downloaded from Zheng et al., 2016 and converted to the bigWig format using ‘bedClip’ and ‘bedGraphToBigWig’ from UCSC Genome Browser database. ‘multiBigwigSummary’ from deepTools was used to compute H3K27me3 signal over the DHS and surrounding region.

### Statistical analyses and data visualization

Statistical analyses were implemented with R (http://www.r-project.org/). Pearson’s r coefficient was calculated using the ‘cor’ function with default parameters. Figures 2b and 4d were generated with R function ‘heatmap.2’. Figures 3d, 4c, 5a–d were generated with R function ‘pheatmap’. Figures 1b and 3b were generated using ‘computeMatrix’ and ‘plotHeatmap’ function in deepTools ^51^. Position-wise coverage of the genome by sequencing reads was determined by normalizing to the total unique mapped reads in the library using macs2 v2.1.0 ^56^ and visualized as custom tracks in the IGV genome browser.

### Known imprinting gene information

Known imprinting information was downloaded from http://www.geneimprint.com/site/genes-by-species.Mus+musculus.

### Code availability

A customized pipeline was used to split the hybrid RNA-seq data to their parental origin based on SNP information. The code can be found at https://github.com/lanjiangboston/UniversalSNPsplit.

### Data availability statement

All the liDNase-seq and RNA-seq datasets generated in this study were summarized in Table S15 and deposited at GEO database under accession number GSE92605. Sperm liDNase-seq datasets were from a previously publication (GSE76642) ^7^. WGBS datasets for sperm and GV oocytes were downloaded from http://www.nodai-genome.org/mouse.html?lang=en ^9^. H3K27me3 ChIP-seq datasets of sperm, MII oocytes, and SNP-tracked maternal and paternal alleles of 1-cell embryos were downloaded from a previous publication (GSE76687) ^11^.

## Extended Data

## Supplementary Material

Table S15

Table S14

Table S12

Table S11

Table S13

Table S10

Table S9

Table S7

Table S8

Table S6

Table S5

Table S4

Table S3

Table S1

Table S2
